# The popliteofibular ligament: a cadaveric ultrasound study

**DOI:** 10.1007/s00256-021-03813-9

**Published:** 2021-06-19

**Authors:** Przemysław A. Pękala, Ewa Mizia, Mitchell R. Mann, Ilona Wagner-Olszewska, Marcin Mostowy, Grzegorz Tatoń, Marcin Domżalski

**Affiliations:** 1 Department of Anatomy, Jagiellonian University Medical College, Kraków, Poland; 2 Faculty of Medicine and Health Sciences, Andrzej Frycz Modrzewski Kraków University, Kraków, Poland; 3 Department of Orthopedics and Trauma Medical, University of Łódź, Veteran’s Memorial Hospital, Łódź, Poland; 4 Department of Biophysics, Jagiellonian University Medical College, Kraków, Poland

**Keywords:** Popliteofibular ligament, PFL, Ultrasonography, Posterolateral corner, Arcuate sign

## Abstract

**Objective:**

The popliteofibular ligament (PFL) is an important stabilizer of the knee found within the posterolateral corner (PLC) of the joint. Injuries to the PLC can cause substantial patient morbidity. Accurate PFL visualization has been historically challenging, impeding injury diagnosis and treatment. The gold standard for in vivo PFL visualization is magnetic resonance imaging (MRI), but this procedure has slice thickness limitations, is costly, and is subject to longer wait times. Ultrasonographic (US) PFL assessment is a potentially viable alternative to MRI. This study aimed to determine the viability of US PFL assessment.

**Materials and methods:**

Ten fresh-frozen lower limb specimens were evaluated for the presence and morphometric characteristics of the PFL via US using an 18.0-MHz linear transducer. The cadavers were then dissected and reassessed for the presence and morphometric characteristics of the PFLs for comparison with US findings. Moreover, the fracture of the fibular styloid process near the site of the insertion of the PFL (the arcuate sign) was simulated and assessed via US.

**Results:**

The PFL was visualized and measured in all ten knees via both US and cadaveric assessments. There were no statistically significant differences in PFL morphometric characteristics determined via US examination and dissection. The fibular styloid fracture was easily identified in US examination.

**Conclusion:**

US imaging is a viable alternative for accurate and effective assessment of the normal PFL. Moreover, the arcuate sign can be evaluated via US.

**Supplementary Information:**

The online version contains supplementary material available at 10.1007/s00256-021-03813-9.

## Introduction

**Fig. 1:**
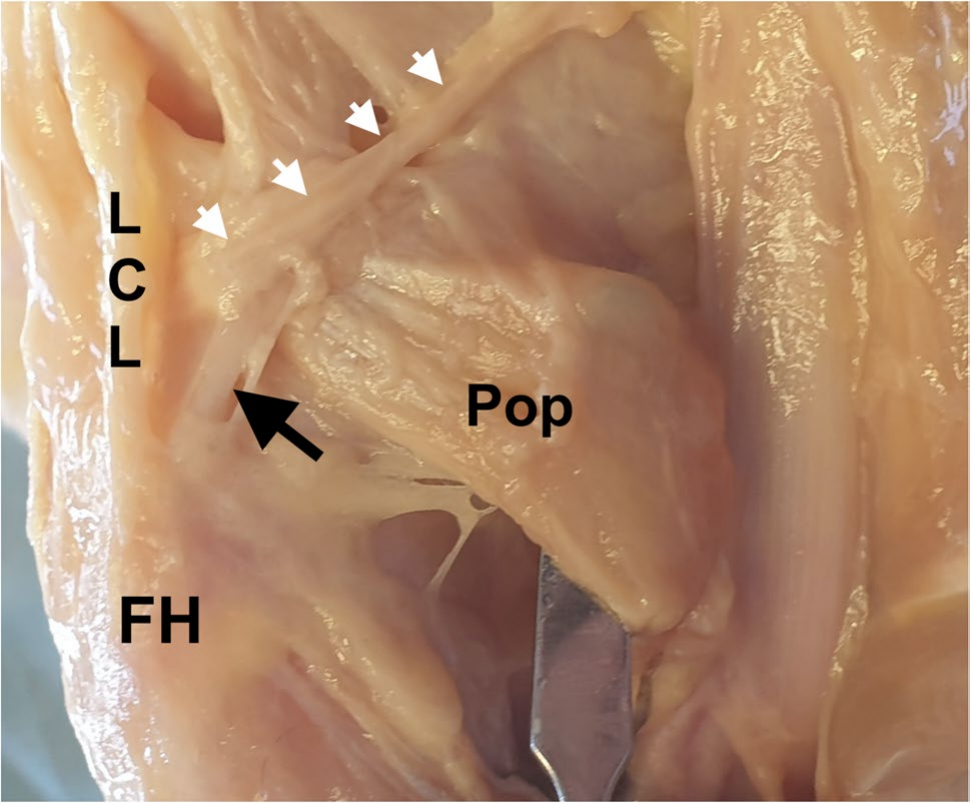
Dissected cadaveric specimen of left knee (posterolateral view, the biceps femoris and gastrocnemius muscle removed). LCL lateral collateral ligament, FH fibular head, Pop popliteus muscle. Black arrow: popliteofibular ligament, white arrows: inferior lateral genicular vessels

The popliteofibular ligament (PFL) is an important stabilizer of the knee, located in the deepest layer of its posterolateral corner (PLC) [1–4]. Working with the popliteus (PT) and lateral collateral ligament (LCL), the PFL assists in preventing external tibial rotation and posterior translation, as well as varus angulation of the knee joint [1]. The PFL spans from its point of origin from the PT, descending to the medial edge of the head of the fibula [5, 6] (Fig. 1). The PFL is consistently strong and thick, with a cross-sectional area similar to that of the LCL but with a flatter shape [5].

The PLC is among the most anatomically complex regions of the knee joint. Due to this complexity and the varied presence and morphology of its structures, the accurate and comprehensive characterization of the PLC has historically been difficult. Accordingly, injuries to such structures may be overlooked by physicians. However, in recent decades, the characterization of the PFL has improved as a result of advancements in imaging techniques and a more detailed account of PLC anatomy [3, 7–11]. It is estimated that PLC injuries account for approximately 16% of knee injuries and are typically due to trauma directed at the anteromedial face of the hyperextended knee joint, typically in sporting and automobile accidents [10, 12]. When untreated, PLC injuries may cause a significant deterioration in patient quality of life in the form of chronic posterolateral rotatory instability (PLRI), chronic pain, early osteoarthritis, and abnormal gait patterns [5, 13–16]. PFL injuries can be missed during orthopedic evaluations, especially when they accompany ACL injuries. It is noteworthy that undiagnosed PFL insufficiency leads to higher loads on anterior cruciate ligament (ACL) grafts and contributes to the failure of reconstructions [17]. Therefore, PFL examinations should always be performed in case of ACL injury and prior to surgical reconstruction of this ligament.

The current gold standard for PLC and PFL imaging evaluation is magnetic resonance imaging (MRI) [18], performed most successfully via T2-weighted coronal and oblique coronal scans [1, 5]. However, MRI detection is complicated by several factors, including the PFL’s location in the deepest layer of the PLC, morphological variations, and, most importantly, the possibility that the PFL will not be visible on scans due to the partial volume effect [1, 5, 6, 19, 20].

Ultrasonographic imaging (US) is a relatively quick and inexpensive imaging modality that is becoming increasingly useful in orthopedic and sports medicine examinations [21]. The development of this modality has been observable through better scanners, transducers, and advancements in techniques. Through US, physicians have access to real-time cross-sections of patient tissues; the modality is especially useful for the evaluation of superficial soft tissues [22]. Moreover, a change in the angle of the probe and its position can allow for an unlimited number of cross-sections to be obtained at various angles in real-time without any additional effort and time-consuming reconstructions, a feat not possible using MRI [21–23].

The goal of this study was to perform high-frequency US on fresh-frozen cadaveric specimens in order to assess the technique’s usefulness in examining the PFL, ultimately determining its merit as a diagnostic tool for PFL evaluation. Moreover, the authors sought to present the US appearance of the arcuate sign (the fracture of the styloid process of the fibula near the site of the insertion of the PFL) for the first time.

## Materials and methods

### Cadaveric subjects

A total of ten non-paired fresh-frozen lower limbs were assessed in the Poznań Lab Institute. These comprised seven males and three females and seven and three left and right lower limbs, respectively. The average age of the subjects was 77.5 ± 13.1 years. Subjects were acceptable for study if they met the following inclusion criteria: (i) at least 18 years of age, (ii) a lack of previous knee surgeries, and (iii) a lack of lower limb deformities visible on examination. The sample size was decided based on the availability of fresh-frozen cadaveric specimens that met the inclusion criteria.

The study protocol was approved by the local ethics committee. All procedures were performed in accordance with the Declaration of Helsinki. Before experimentation, all limbs were thawed at room temperature.

### Ultrasound assessment

Two physicians (an orthopedic surgeon [E.M.] and an orthopedic surgery resident [P.A.P]) with experience in musculoskeletal US assessed the specimens for the presence of the PFL using a MyLab 25 Gold US scanner with an 18.0-MHz linear transducer (Esaote, Genoa, Italy).

**Fig. 2:**
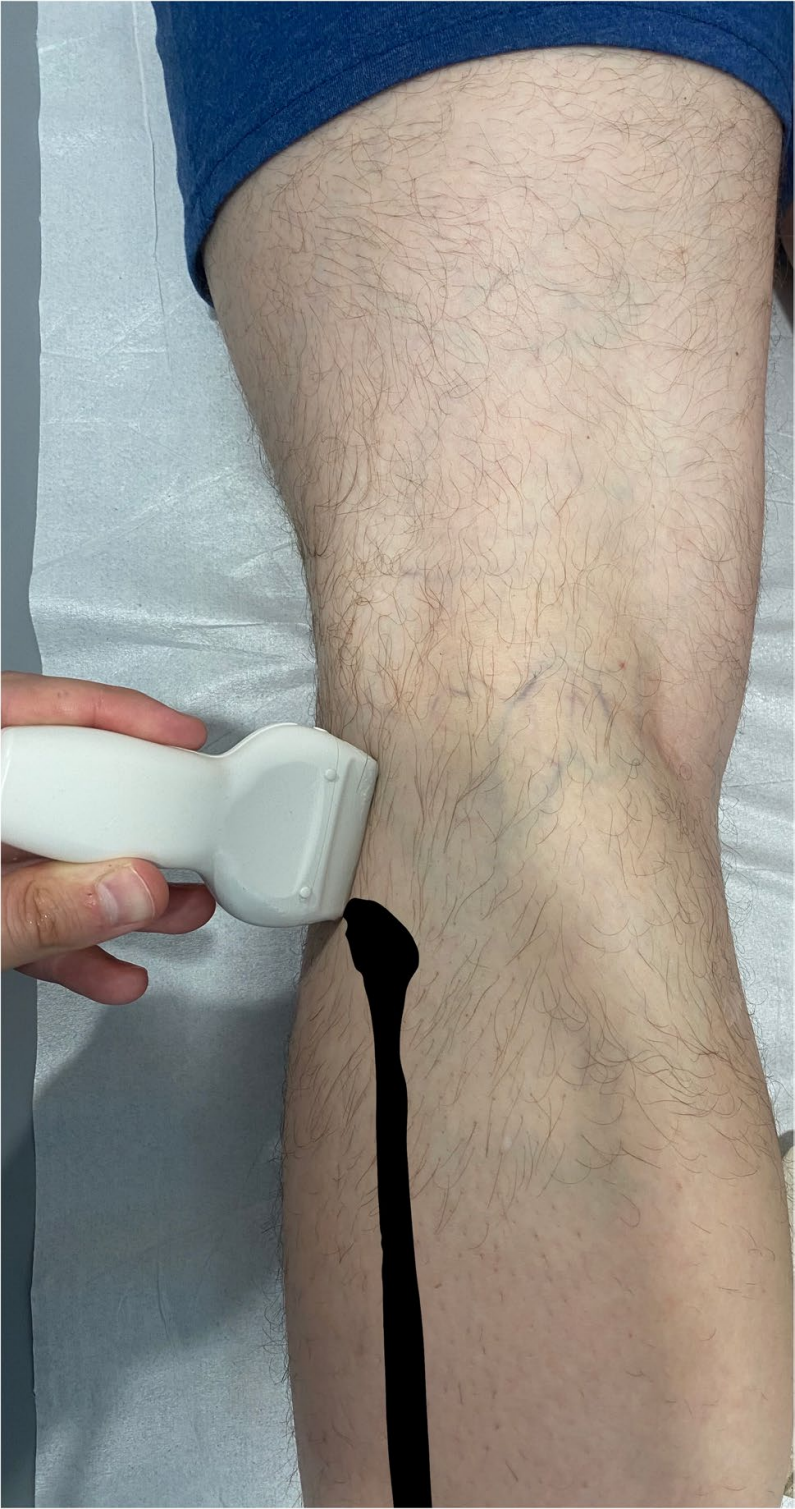
The position (oblique longitudinal orientation) of the US probe used for the popliteofibular ligament (anatomical long axis) visualization. Posterior surface of the left knee, patient is lying in prone position (the fibula marked in black)

Lower limbs were kept in the standard position for popliteal fossa examination (limb extended at the knee joint, prone position). To visualize the PFL, the hyperechogenic cortex of the fibular head was first identified, and the inferior part of the transducer was positioned over this landmark, with the long axis of the transducer being kept parallel to the long axis of the limb. Then, the superior part of the transducer was slowly moved medially with the popliteus tendon cross-section constantly in the field of view (Fig. 2).

**Fig. 3:**
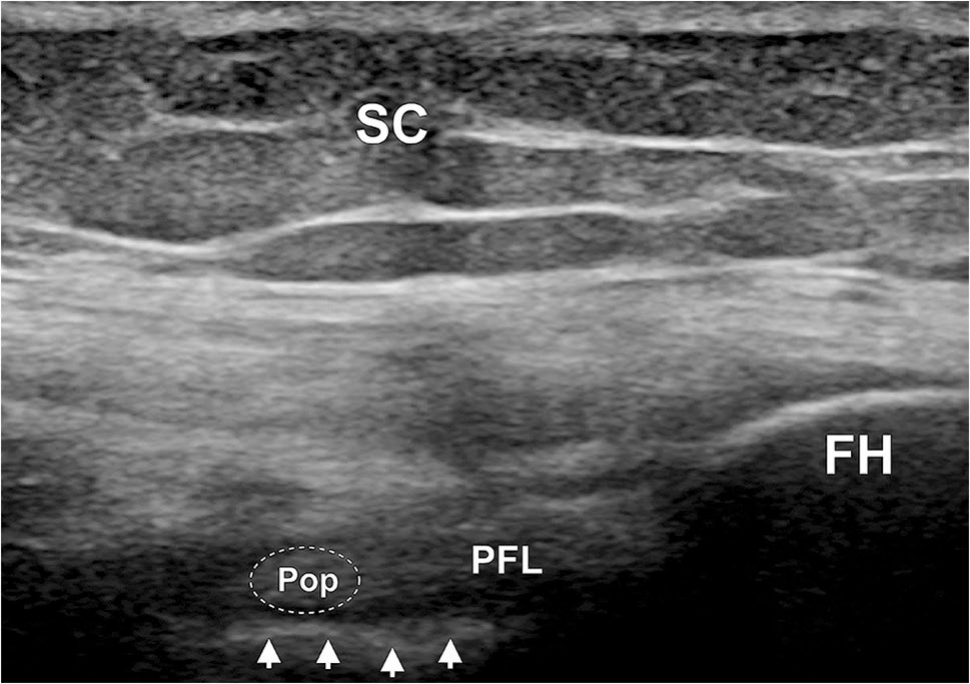
Ultrasound presentation of the popliteofibular ligament (PFL) with its junction to the popliteus tendon (Pop, marked with dashed line) observed in a cadaveric specimen. FH fibular head, SC subcutaneous adipose tissue. White arrows: cortex of the tibia. The US probe is positioned in the oblique longitudinal orientation (in the anatomical long axis of the PFL—superiorly the transducer was medial and inferiorly the transducer was lateral). The proximal direction is to the left and the distal is to the right

The PFL appeared as a fibrillar band extending from the fibular head to the popliteus tendon (joining the muscle in the area near its musculotendinous junction) (Fig. 3).

Moreover, the following measurements were taken during the US examination: (i) length of the PFL (L-US), (ii) width at the insertion to the fibular head–distal end (DW-US), and (iii) width at the junction with popliteus muscle–proximal end (PW-US). In the case of any disagreements, a final decision was made via discussion and consensus among the examiners.

### Cadaveric dissection

Dissections were performed following US examination by two physicians (an orthopedic surgeon [M.D.] and an orthopedic surgery resident [P.A.P]) with experience in anatomical dissections. First, a longitudinal incision was made over the posterolateral aspect of the knee joint. Then, the subcutaneous tissue was dissected, and the biceps tendon was cut from its insertion into the fibular head (Fig. 1). Finally, the PLC structures were carefully dissected using sharp and blunt techniques. The PFL was identified and assessed for its morphological characteristics. The same measurements made via US examinations were performed using an electronic caliper. Appropriate quantitative parameters for cadaveric measurements (CAD) are denoted as L-CAD, DW-CAD, and PW-CAD, respectively. All measurements were recorded by another team member (not involved directly in the measurements) after a consensus was reached among two examiners.

### Arcuate sign simulation

After dissection, the fracture of fibula at the site of the insertion of the PFL (the arcuate sign) was simulated (using osteotome) in one specimen with the knee positioned in the configuration specific for the posterolateral injuries (external tibial rotation, varus angulation, and hyperflexion). The tissues were then moisturized using saline, and dissected layers were put into the previous anatomical position. Finally, a US examination was conducted to document the appearance of the arcuate sign.

### Statistical analysis

Elements of the descriptive statistics (mean, range, SD) were calculated. The results obtained in means of US studies and caliper measurements were compared. The normality of variable distributions was checked by the Shapiro–Wilk test. The *t* test was used to assess the statistical significance of potential differences between L-US and L-CAD as well as between PW-US and PW-CAD. Because DW-US did not perform the normality condition, the two-sample Kolmogorov–Smirnov test was used to test DW-US and DW-CAD dependence.

In order to determine the precision of the US results in comparison to true PFL dimensions, linear regression was performed, and the Pearson correlation coefficient was calculated. The *F*-test was used to test the equality of variances.

All calculations were performed using SPSS software version 25 (IBM, USA). A *p-*value of < 0.05 was considered statistically significant.

The AQUA checklist [24] was followed throughout this study (Electronic Supplementary Material).

## Results

### Frequency of PFL visualization

The PFL was identified in all specimens (10/10, 100.0%) using both US and cadaveric dissection. The PFL was presented as a fibrillar sheet-like band connecting the styloid process of the fibula and the popliteus muscle near its musculotendinous junction, lying in the deep portion of the PLC, just superficial to the cortex of the tibia (Fig. 3).

It is worth emphasizing that, for in vivo US studies (normal clinical settings), the inferior genicular artery (located just superficial to the PFL) is a perfect landmark for PFL positioning (Fig. 1). Moreover, Doppler imaging can be used to better visualize this vessel in vivo.

### PFL morphometrics

The mean length (L) of the PFL in US investigations was 17.3 ± 3.9 mm and was 17.9 ± 3.3 mm in cadaveric dissections.

The mean PFL widths at the insertion to the fibular head (DW) and at the junction with the popliteus muscle (PW) were 11.1 ± 4.7 mm and 11.5 ± 4.0 mm in the cadaveric investigations and 11.3 ± 6.0 mm and 11.7 ± 4.4 mm in the US evaluations, respectively (Table 1).

**Table 1:** The descriptive statistics of quantitative data obtained in ultrasonographic measurements (L-US, PW-US, and DW-US) and in caliper measurements after PFL dissection (L-CAD, PW-CAD, and DW-CAD). *L* PFL length, *DW* PFL width at the insertion to the fibular head–distal end, *PW* width at the junction with popliteus muscle–proximal end. All distances are expressed in millimeters

	Age	L-US	L-CAD	PW-US	PW-CAD	DW-US	DW-CAD
Mean	77.5	17.3	17.9	11.7	11.5	11.3	11.1
SD	13.1	3.9	3.3	4.4	4.0	6.0	4.7
Median	76	15.7	17.5	11.2	11.6	8.1	11.0

According to the *t*-test results for L and PW and the Kolmogorov–Smirnov test assessment for DW, there were no statistically significant differences between the measurements taken during US examinations and cadaveric dissections.

The results of linear regression and Pearson coefficient calculations are recorded in Table 2.

**Table 2:** The results of linear regression and Pearson coefficient calculations. The L-US, PW-US, and DW-US were fitted as functions of the L-CAD, PW-CAD, and DW-CAD, respectively. *L* PFL length, *DW* PFL width at the insertion to the fibular head–distal end, *PW* width at the junction with popliteus muscle–proximal end, *US* the ultrasonic measurement, *CAD* caliper measurement after dissection

	(L-US) = b ∙ (L-CAD) + a	(PW-US) = b ∙ (PW-CAD) + a	DW-US = b ∙ (DW-CAD) + a
b	1.0	0.67	1.0
Db	0.2	0.31	0.3
a [mm]	− 1.3	4.0	− 0.1
Da [mm]	3.4	3.8	3.3
r	0.892	0.610	0.801

### Arcuate sign evaluation

**Fig. 4:**
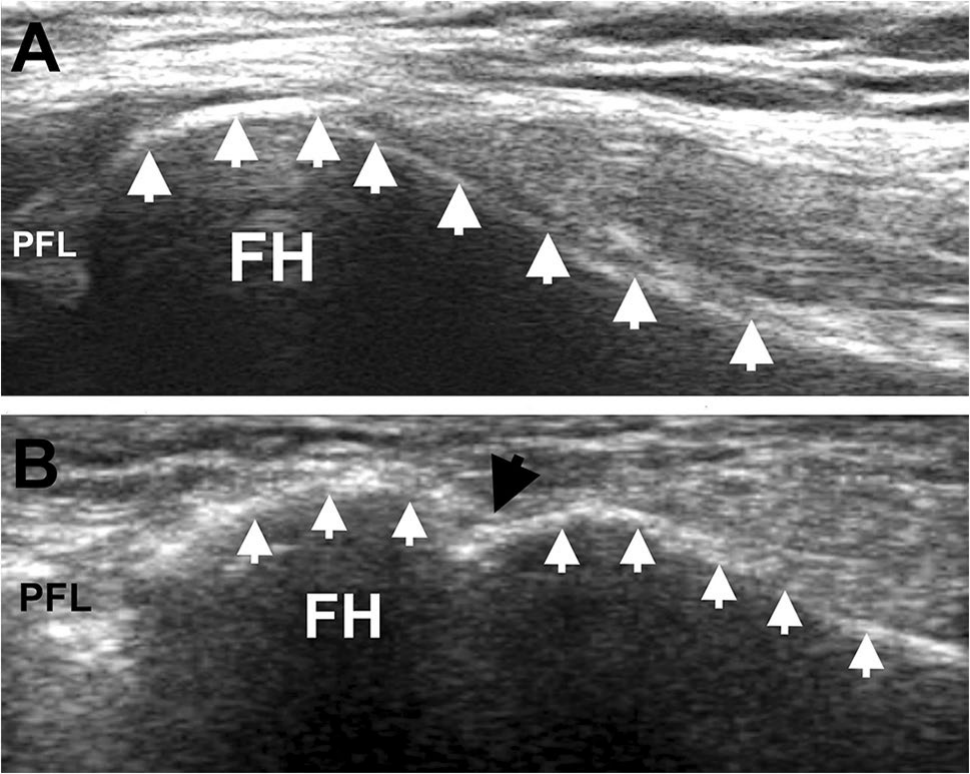
Ultrasound presentation of **A** the normal cortex of the fibular head and **B** the arcuate sign (fracture of the fibular styloid process distal to the popliteofibular ligament insertion). FH fibular head. White arrows: hyperechoic signal of the cortex of the fibular head, large black arrow: disruption in the cortex of the fibular head caused by the fracture of distal to the insertion of the popliteofibular ligament. The US probe is positioned in the long axis of the fibula, over its head. The proximal direction is to the left and the distal is to the right

The fracture of the fibular styloid process near the site of the insertion of the PFL (the arcuate sign) was clearly visualized via US and documented (Fig. 4). It was best visualized when the long axis of the probe was parallel to the fibular long axis. The fracture presented as a hypoechogenic gap in the hyperechogenic cortex of the bone. This contrasts with the normal US appearance of the head of the fibula, which presents as an uninterrupted linear hyperechogenic signal along the proximal fibula (Fig. 4).

## Discussion

The principal aim of this study was to show that US assessment is a viable strategy for identifying the PFL for the purpose of examining the integrity (e.g., the presence of tears or avulsions), structure, and size of the ligament. This study showed that the PFL could be visualized in all assessed knees during US examinations with the use of a high-frequency US probe. The US visualization of the PFL was verified via cadaveric dissection in all cases. Moreover, no significant statistical differences were noted in the morphometric properties of the PFL when assessing the ligament via US and dissection.

Additionally, this study provides a unique descriptive value for the arcuate sign, which has never been described via US. US assessment clearly visualized the fracture near the insertion of the PFL into the fibular head (Fig. 4). Together, these findings indicate that US can be used as a fast and cost-effective point-of-care diagnostic tool when assessing PFL anatomy. However, further studies are required to fully investigate the pathological changes of the PFL via US examination.

The PFL is considered a constant structure within the human knee. However, its prevalence is lower among MRI studies than in cadaveric investigations [20]. This is most likely due to gaps between established MRI examination cross-sectional slices (partial volume effect) that miss the thin PFL; as a result, the PFL (a sheet-like structure) may be excluded from MRI cross-sections, making its assessment difficult through this modality [1, 5, 6, 20]. As injury to the PFL may lead to severe morbidity and is a cause of failure of ACL reconstruction surgery, it is important to fully assess this structure in the case of complex knee injuries [17]. Ultrasound, due to its ability to obtain variable cross-sections in real-time, can be considered a useful diagnostic tool for orthopedic surgeons assessing and treating knee trauma [11, 25].

Currently, US is an underexplored method for assessing the PLC and its injuries, having only been demonstrated previously by Sekiya et al. [11, 25], Barker et al. [26], and De Maeseneer et al. [27]. However, to the best of the authors’ knowledge, there are currently no studies presenting statistical analyses comparing US morphometric measurements of the PFL with those obtained via cadaveric dissection.

The use of US to assess the PLC was performed by Sekiya et al. in 2002 [11]. The authors used 10- and 12-MHz linear transducers to assess seven cadaveric knees. Using bony landmarks as a point of reference, the authors successfully visualized all PLC structures, including the PFL, which they described as being attached to the PT and fibular head and adjacent to the posterior tibial cortex when viewed along a sagittal plane. Barker et al. [26] further explored sonographic imaging of the PLC. The authors used a 12.5-MHz linear transducer to view the PFL within live patients during knee flexion, observing the structure as being linear and hypoechoic, and spanning between the lateral aspect of the popliteus muscle and the medial aspect of the fibular apex. In 2010, Sekiya et al. [25] followed up on their previous work, this time using both static US and a dynamic US stress test on 16 patients. The authors noted an overall accuracy of PFL visualization of 69% (11/16 knees). The patients were further divided by clinical status, with 12 requiring surgical intervention and four not needing further treatment. Their US determination of PFL clinical status showed sensitivity, specificity, and positive and negative predictive values of 67 (8/12), 75 (3/4), 67 (8/12), and 75% (3/4), respectively. The authors attributed the lower accuracy of PFL visualization to the ligament’s depth within the PLC, particularly when compared to the LCL, which had an accuracy of 88% (14/16). When using dynamic US stress tests to predict injuries to the posterolateral knee, they noted an accuracy level of 88%, as well as sensitivity, specificity, and positive and negative predictive values of 83, 100, 100, and 75%, respectively, further displaying the potential for US as a diagnostic tool for PLC injuries. In their study, Sekiya et al. [25] used 7–12-MHz linear transducers; we believe that our higher percentages of PFL visualization are most likely due to the use of newer, higher frequency (18.0 MHz) linear transducers and scanners, which allow for better visualization of superficial soft tissues.

The results of our statistical analysis did not show significant differences between the measurements taken during US examinations and cadaveric dissections (CAD). However, this does not mean that both methods are always able to deliver similar quantitative results. This becomes apparent after an analysis of the data within Table 2. Both methods give the same results, provided that three conditions are fulfilled: (i) there is a high-quality linear model fitting to the considered dependencies (*r* ≈ 1), (ii) the slopes (“a” parameter) are close to one, and (iii) the intercept (“b” parameter) is close to zero. These conditions are accomplished relatively well for the L and DW measurements, but not for PW. This is likely caused by the anatomy of the PFL near its junction with the PT, making it difficult to measure its width at the exact same level repeatedly, in contrast to its attachment to the fibular head (the clear point, where hyperechoic bone is located). The problem of making PFL measurements, especially in its proximal part, while using US imaging should be addressed more precisely in further studies.

The authors of this study strongly encourage the use of constant anatomical landmarks in order to identify the PFL. Noteworthy is the fact that the PFL is crossed by the inferior lateral genicular vessels, which run just superficial to it (Fig. 1) and can be easily visualized in vivo via Doppler imaging. It is important not to confuse the PFL with the fabellofibular ligament, which is located superficial to the inferior lateral genicular vessels. Also, the fibular styloid process is a constant hyperechoic landmark that can be considered an easy starting point in PFL visualization from which the probe can be slowly moved superiorly to find the popliteus tendon. Moreover, to improve PFL visibility and confirm its function, the tibia can be externally rotated, with the knee joint subjected to varus stress and hyperextended (pad under the anterior thigh when the patient is lying prone).

Despite the fact that the PFL is believed to be a constant or rarely absent structure, it is crucial that physicians are aware of its anatomical variations and know the limitations of US evaluation. The different appearances of the PFL were described in the cadaveric study by Zeng et al. [28]; they reported that, although the PFL was constantly present in their specimens, in 12.3% of cases, it was thinner and fascia-like. We hypothesize that in such individuals the visualization of the PFL could be challenging or even impossible via US.

The avulsion fracture of the fibular styloid process by the PFL or other anatomical structures attaching to it, such as the fibular collateral ligament or biceps femoris tendon, is known as the arcuate sign. It is commonly associated with multi-ligamentous ruptures of the knee (e.g., in the PLC, as well as the ACL and PCL) and has been described in the literature using both x-ray and MR imaging [6, 27, 29, 30]. However, this study provides its first description using US. The authors believe that screening examinations of the cortex of the head of the fibula along its long axis in cases of complex knee trauma can contribute to the fast, easy, and effective identification of this clinically important fracture.

This study was limited by the technical capabilities of the ultrasound scanner. However, a high-quality linear probe (18.0-MHz) was used to optimize the evaluation of the PFL. It is noteworthy that US examination is a subjective diagnosis modality, and its interpretation can vary among physicians performing scans. However, all observations in this study were confirmed by consensus among two examiners to reduce observer bias. The number of assessed specimens can be considered a limitation for this investigation, but it was sufficient to reach the threshold required for statistical analysis. Moreover, the appearance of the assessed tissues in cadavers may be different than in living humans. However, for this type of modality, only the employed study design could have allowed us to verify our hypothesis, and particular care was taken to obtain an environment as close to the human body as possible (fresh-freezing and performing examinations just after thawing). Lastly, the cadavers used for this study were mostly elderly and had signs of osteoarthritis. However, this only made visualization of the PFL more challenging, and examinations of the younger subjects should be easier to interpret. More studies, particularly ones of a clinical nature assessing larger populations, should be performed in the future to evaluate the effectiveness of US in PLC injury diagnosis.

It is noteworthy that some factors, such as the presence of hematomas, edema, soft tissue abnormalities, and other pathological phenomena, pose complications in tissue assessments and must be considered when interpreting scans.

In conclusion, this study showed that high-frequency US can be considered a fast, cost-effective, and readily available method for point-of-care visualization of the PFL. Moreover, it enables for the dynamic assessment of this structure at various angles, theoretically allowing for unlimited cross-sections. When conducted by physicians experienced in knee US, PFL visualization (without measurements) adds approximately 1 min to a standard examination. Physicians performing an ultrasound examination of the PFL must be aware that it requires specialized experience. Finally, small fractures of the fibular styloid process can be clearly visualized via US.

## Supplementary Information

ESM 1 (DOCX 30.9 kb)

## Data Availability

Not applicable.
